# Assessment from a Biopsychosocial Approach of Flight-Related Neck Pain in Fighter Pilots of Spanish Air Force. An Observational Study

**DOI:** 10.3390/diagnostics12020233

**Published:** 2022-01-19

**Authors:** Luis Espejo-Antúnez, Carlos Fernández-Morales, Juan Manuel Moreno-Vázquez, Fernando Blas Tabla-Hinojosa, María de los Ángeles Cardero-Durán, Manuel Albornoz-Cabello

**Affiliations:** 1 Department of Medical-Surgical Therapeutics, Faculty of Medicine and Health Sciences, University of Extremadura, Av. Elvas, S/N, 06006 Badajoz, Spain; luisea@unex.es (L.E.-A.); m.angeles.cardero@gmail.com (M.d.l.Á.C.-D.); 2 Department of Biomedical Sciences, Faculty of Medicine, University of Extremadura, Av. Elvas S/N, 06006 Badajoz, Spain; jmmoreno@unex.es; 3 Head of Medical Staff of Talavera’s Air Base, Spanish Air Force, 06071 Badajoz, Spain; fernandotabla@gmail.com; 4 Department of Physical Therapy, Faculty of Nursing, Physical Therapy and Podiatry, University of Seville, C/Avicena, 6, 41009 Seville, Spain; malbornoz@us.es

**Keywords:** neck pain, evaluation study, pilots, disability

## Abstract

Flying on fighter aircraft is the only human activity that exposes the body to acceleration levels for long periods of time. In this sense, the regular exposure to G forces has been related to a high incidence of flight-related neck pain. The aim is to evaluate flight pilots of the Spanish Air Force (instructors vs. students) diagnosed with flight-related neck pain from a biopsychosocial perspective. Eighteen fighter pilots with flight-related neck pain were divided into two groups: instructor fighter pilots (*n* = 7) and student fighter pilots (*n* = 11). The Neck Disability Index (NDI), Cervical Range of Motion (CRoM), Pain Pressure Threshold (PPT), cervical repositioning error, and myoelectric activity were evaluated. Cervical flexion, extension and left and right rotation showed a reduced range of motion in both groups with respect to the normative values of the healthy population. There were no statistically significant differences between the groups (*p* ≥ 05). The correlational analysis showed a strong association between the NDI and CRoM of the left rotation (β = −0.880, *p* = 0.002). The NDI also had a positive association with the pilot’s age (β = 1.353, *p* < 0.01) and the number of flight hours (β = 0.805, *p* = 0.003). In conclusion, the Cervical Range of Motion at the left rotation seems to determine the perceived degree of disability in both the instructors and students. This factor could be influenced by the number of flight hours and accumulated experience as an F-5 fighter pilot.

## 1. Introduction

Flying on fighter aircraft is the only human activity that exposes the body to acceleration levels for long periods of time, sometimes even higher than that of the Earth’s gravity (G) [1]. During flights on fighter aircraft under high G-forces, the spine is exposed to heavy loads [2], with the consequent risk of developing flight-related neck pain [2]. This musculoskeletal disorder refers to significant neck pain that occurs during or within 48 h after the flight. It does not refer to pain that is due to other activities or causes [3]. It is a common problem for military pilots, with a reported prevalence of 66% for all Royal Air Force aircrew and 70% for UK fast-jet pilots [4]. In the general population, the prevalence of nonspecific mechanical cervical pain (NSMNP) is associated with increased age [5,6]. However, the risk of aircrew neck pain is multifactorial. Some of these factors include the regular exposure to high G-forces [7] or the influence of helmet-mounted system factors combined with a higher operational tempo (mission intensity, duration, and frequency) [3].

Previous studies [8,9,10,11,12] showed that pilots flying on aircraft with a greater capacity to withstand G-forces had a higher incidence of neck pain. This fact has been observed in different countries around the world, such as in the United States [7,8,9], Australia [10] and Japan [11]. In Europe, Lange et al. [13] observed that at least the 83% of the Danish Air Force pilots that were surveyed reported neck pain during the flight or at later times over the last year.

Despite this, a recent NATO document reported the possible causal factors associated with flight-related neck pain, such as human factors, body-borne equipment, aircrew behaviors, aircraft workspace or organization [3]. In this regard, Keogh et al. [14] analyzed the interference of experimental pain on the processing of tasks that require the engagement of higher-order cognitive centers. These authors showed that even though it was thought that no effects of pain on the processes would underpin complex tasks, there were indirect changes in cognition. On the other hand, Casner et al. [15] analyzed the technical skills acquired by pilots due to unexpected events in flight conditions, e.g., aerodynamic failures, dangerous weather situations, concussions, etc.

Among military aircrew, few studies have specifically analyzed not only the physical symptoms associated with flying activities (e.g., neck muscle strain) but also the social or psychological effects that reveal feelings or concerns that have an impact on non-work time [16,17].

Although the need to quantify the workload in terms of military crews’ neck pain has been highlighted [17], there are limited studies that analyze the psychological and social factors in addition to the physical symptoms (biopsychosocial model). Due to the benefits reported by different physiotherapy modalities in the military population [18], it seems necessary to detect the physical, psychological and functional parameters related to other ailments in this population such as flight-related neck pain. The aim of this study was to design a reliable assessment protocol based on the biopsychosocial model of military pilots with flight-related neck pain, and to understand the possible associations as a function of age and flight experience.

## 2. Materials and Methods

### 2.1. Study Design

This was a comparative-correlational study based on a cross-sectional study. This study was supervised by the Bioethics Committee of the University of Extremadura, with the ethics approval number 54/2020 registered in ClinicalTrials.gov (NCT:04396691), and it was also performed in accordance with the Strengthening the Reporting of Observational Studies in Epidemiology (STROBE) statement [19]. Regarding ethical procedures, all participants signed an informed written consent to participate in this study.

### 2.2. Participants

**Figure 1:**
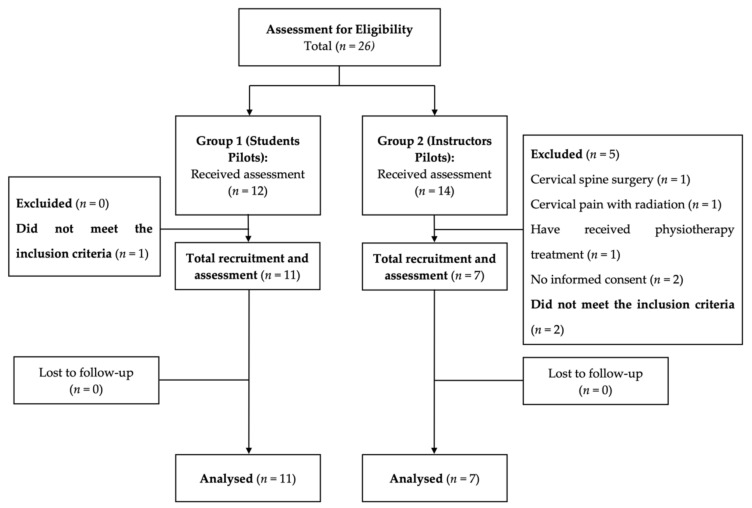
Flow diagram of pilot recruitment.

An initial, potentially eligible sample of 26 fighter pilots (the total staff of instructors and students for the academic year 2020–2021) and adult volunteers were recruited. The inclusion criteria were: (i) flight pilots (male and female) who, at the time of the assessment, were an instructor or student attached to the 23th Wing of Talavera Air Base, Spanish Air Force (SAF), Badajoz. The student pilots were characterized by the fact that they were undergoing training at the General Air Academy. At the time of the study, they were in their fifth year of training. At the end of their training period at the General Air Academy, they will join the Air Force of the Spanish Ministry of Defense as student pilots. In contrast to the students, the instructor pilots, in addition to having completed their studies leading to a degree in Aeronautical Engineering, are part of the Air Force’s in-service staff as military fighter and attack pilots; (ii) flight pilots diagnosed with flight-related neck pain according to the International Classification proposed by a NATO panel of experts [3]; (iii) a minimum perceived pain of 3/10 on the Visual Analogue Scale (VAS) in the early-morning assessment; (iv) scores of ≥5 points on the Neck Disability Index (NDI), and a cervical-repositioning error of ≥4.5° [20]. The exclusion criteria were: (i) cervical pain with radiation to the upper limbs and/or radiculopathy; (ii) cervical spine surgery with or without the presence of a metal implant; (iii) having received physiotherapy or any other routine medical care six weeks prior to data collection; (iv) being involved in ongoing medical–legal conflicts. Finally, the total sample consisted of 18 male fighter pilots (instructors and students) (Figure 1). The recruitment period went from 1 November 2019 to 31 January 2020. Figure 1 provides a flow diagram of subject recruitment during the study.

### 2.3. Assessment

Data collection was recorded in supine and sitting positions, in the early morning by an physiotherapist evaluator with more than 15 years of experience [13].

#### 2.3.1. Primary Outcomes

Cervical Disability: Cervical Disability was evaluated using the validated Spanish version of the Neck Disability Index (NDI) [21]. This questionnaire consisted of 10 sections, 4 of which were related to subjective symptoms (pain intensity, headache, ability to concentrate and sleep quality) and the other 6 were related to basic activities of daily life (personal care, ability to lift weights, reading, work, driving, leisure activities and leisure time). The NDI has been shown to be valid and reliable for measuring pain and cervical disability (Cronbach Alpha: 0.944; (ICC(95%CI))]:0.88 (0.80, 0.93)) [22]. It is the most used scale for neck pain and disability, having been previously used in fighter pilots [23].

Each item of this self-completed questionnaire presented six possible answers. Scores ranged from 0 (no disability) to 5 (complete disability) points. The total score (maximum 50 points) was calculated by adding up the responses for each item. The interpretation of the questionnaire was classified as follows: “no disability” (NDI < 10% of maximum total score), “mild” (NDI between 10% and <30%), “moderate” (NDI between 30% and <50%), “severe” (NDI between 50% and <70%) or “very severe” (NDI >= 70%) degrees of disability.

Cervical Range of Motion (CRoM): The pain-free active CRoM was defined as the maximum amplitude that the patient could actively achieve at a standardized, upright sitting position without pain. Each participant sat in a chair with a backrest with their feet flat on the floor and their arms hanging alongside their body, avoiding compensation with other body regions [24]. The evaluator measured the sagittal (flexion/extension), frontal (right/left lateroflexion) and transverse planes (right/left rotation) using an individual head goniometer (Enraf-Nonius© BV, Rotterdam, The Netherlands) as indicated by Nagai et al. [24]. Every physiological movement was repeated three times with 30 s of rest [25]. The goniometer is a valid and reliable measuring instrument used in the cervical region [26]. The obtained values were referenced with the results reported by Kauther et al. [27] in a sample of 4293 young male adults with chronic neck pain (average age: 27.08).

#### 2.3.2. Secondary Outcomes

**Figure 2:**
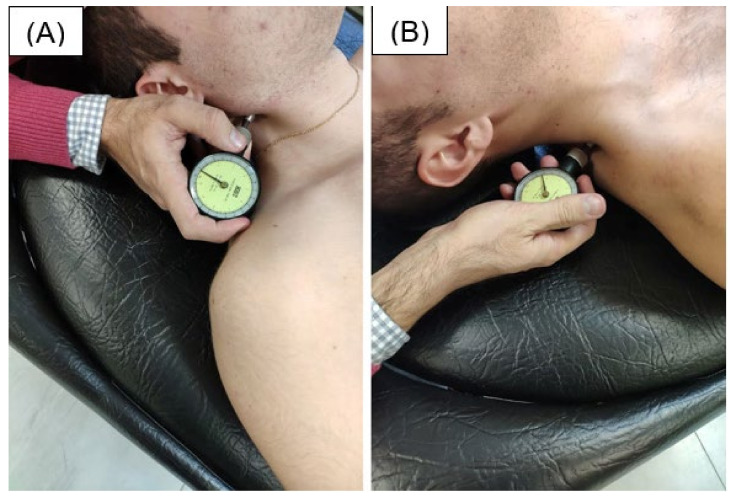
Sternocleidomastoid (**A**) and upper trapezius (**B**) algometry.

Pain: Pressure Pain Threshold and pain intensity: The pain perceived during manual palpation of the Myofascial Trigger Points (MTrPs) was evaluated. MTrPs are defined as hypersensitive regions in taut bands of a skeletal muscle that are painful upon stimulation (i.e., palpation) and elicit referred pain. MTrPs of the sternocleidomastoid (SMC) muscle, upper trapezius and elevator muscle were bilaterally evaluated by the evaluator who also measured the other variables (a physiotherapist with more than 15 years of experience), coinciding with the characteristics indicated by Lange et al. [13] (Figure 2). The MTrPs (active and latent) were identified following the original diagnostic criteria and the subsequent revision of the diagnostic criteria described by Simons [28]. Barbero et al. [29]. reported moderate to high reliability (ICC = 0.62 (95% CI: 0.30–0.81, and ICC = 0.81 (95% CI: 0.61–0.91) when MTrP locations in the upper trapezius muscle’s identification was performed by an experienced physiotherapist.

Then, a mechanical-pressure algometer (Baseline^®^ Fabrication Enterprise. Inc. P.O Box 1500 White Plains, NY, USA) with a 1 cm^2^-area contact head was used to measure the PPT of the Myofascial Trigger Points (MTrPs). Linde et al. [30] showed a strong positive, linear relationship between the rate of pressure application and PPT, as measured by an algometer (Pearson’s correlation coefficient = 0.78 ± 0.19). The pressure algometer was aligned perpendicularly on the skin over the clinically identified MTrP site while pressure was consistently applied downward onto the MTrP site [30]. A gradual and continuous pressure was applied perpendicular to the muscles, at approximately 1kg/cm^2^ (Figure 2).

Three PPT measurements were taken with a 2 min rest interval between trials. The mean of the 3 trials was calculated and used for the analysis. The participants were asked to rate pain intensity in response to manual palpation of MTrPs using a 10 cm Visual Analogue Scale (VAS). This scale has been proposed to measure pain perceived by pilots, ranging from 0 (“no pain at all”) to 10 (“worst imaginable pain”) [3].

Myoelectric activity: Myoelectric activity was measured by surface electromyography (sEMG) according to the recommendations of the NATO expert panel [3]. A protocol similar to that established in previous studies was used [16,31]. The sEMG recordings were taken using mDurance^®^ system (mDurance Solutions SL, Granada, Spain). It is a portable and low-cost EMG system, which has proved to be a valid tool to measure muscle activity during dynamic contractions [32]. The system consists of a Shimmer3 EMG unit (Realtime Technologies Ltd., Dublin, Ireland) and a bipolar sEMG sensor for the acquisition of superficial muscle activity. Each Shimmer sensor is composed of two sEMG channels, which register the electrical signal via Bluetooth with a sampling rate of 1024 Hz. Shimmer applies a bandwidth of 8.4 kHz, the EMG signal resolution is 24 bits, and the overall amplification is 100–10,000 V/V.

As sEMG is recommended for the noninvasive assessment of muscles SENIAM [33], the electrode-placement areas were shaved if necessary and disinfected with 70% alcohol. Next, five disposable, bipolar, self-adhesive electrodes with pre-gelled surface discs and an active diameter with dimensions 41 mm × 21 mm, diameter 10 mm (Ag/AgCl, Blue Sensor N-00-S, Medicotest A/S, Olstykke, Denmark) were placed pairwise (with an interelectrode distance of 21 ± 1 mm) on the muscle belly parallel to the muscle fibers. Myoelectric activity was bilaterally recorded from two muscles: the anterior neck (the lower part of the sternocleidomastoid muscle belly) and the upper shoulders (upper trapezius muscle bellies, midway between the occiput and the acromion).

A reference electrode was placed around the bone rim of the acromion.

Firstly, participants were instructed through pre-registration tests before myoelectric-activity recordings were made. Pre-recording tests consisted of the subject pushing their heads against manual resistance in extension, as indicated by Pousette et al. [16]. Subjects were comfortably seated in the gondola’s flight seat, with their back against the seat and their trunk and shoulders fixed to a rigid seat back using two straps placed along both shoulders, and the elbows with a flexion of approximately 60°. The participants were placed in a neutral cranio-cervical position, then they were instructed to push with increasing force up to a maximal voluntary force and hold for 3 s in order to avoid injury and minimize the risk of dynamic contractions. Three trials were done with 1 min rest in between each other in order to enhance maximum voluntary contraction (MVC) stability [16].

Then, three trials (with a duration of 1 s for each attempt) at 15–30% MVC were performed, as proposed by Calamita et al. [34], and the mean of the two highest trials was defined as the MVC.

Kinesiophobia: The Spanish version of the Kinesiophobia Tampa Scale (TSK-11) was used. This subjective questionnaire contained 11 items designed to assess the patient’s fear of moving and re-injury. The score ranged from 11 to 44 points. Each item was associated with a 4-point Likert scale (1 = “strongly disagree”, 4 = “strongly agree”). Higher scores corresponded to a greater fear of pain, movement and injury. The Spanish version of the TSK-11 has shown good reliability and validity (Cronbach alpha 0.79) [35]. A higher score indicates higher levels of kinesiophobia.

Catastrophizing Pain: Catastrophizing is a cognitive factor that involves an exaggerated negative appraisal toward pain stimuli and pain experience [36]. The Pain Catastrophizing Scale (PCS) is a self-administered scale (Likert scale) of 13 items and one of the most used and reliable (Cronbach alpha 0.79) [37] to assess the catastrophizing of pain. In it, subjects refer to their past painful experiences and indicate the degree to which they experienced each of the 13 thoughts or feelings. The score ranges from 0 (never) to 4 (always).

The total score reflects the subject’s level of pain catastrophism (a higher score means worse result).

### 2.4. Statistical Analysis

Data analysis was performed using SPSS version 20.0 (SPSS Inc., Chicago, IL, USA). A descriptive analysis of each of the variables was performed. The normality of the variables was evaluated using the Shapiro–Wilk test; consequently, parametric (*t*-student test or Welch test) or non-parametric (Mann–Whitney or Wilcoxon test) tests were applied. Data are reported as mean ± SD. The demographic and clinical variables of the groups at baseline were compared using the chi-square test for categorical data and the independent-samples *t*-test for quantitative data.

The correlation study between different variables was performed using linear correlation analysis (Spearman correlation coefficient) and linear regression model adjustment. It was checked whether the correlations established were statistically significant by adjusting the corresponding linear regression models and analyzing the corresponding analysis tables of the associated variances. The significance level was established at *p* < 0.05.

### 2.5. Sample Size Estimation

G*power 3.1 software was used to calculate the sample size required to detect changes in the primary outcome (CRoM). Assuming an effect size (*p*) of 0.55 for correlation and point-biserial model, an alpha level of 0.05 and a power of 80 %, a total sample size of 16 participants was estimated. The final sample was of 18 participants.

## 3. Results

There were no significant baseline differences between the groups in any of the sociodemographic characteristics or biopsychosocial parameters (*p* ≥ 0.05 for all comparisons), except for age and number of flight hours (Table 1 and Table 2). The ages were between 21 and 34 years old, with a mean age of 34 ± 6.1 among instructors and of 21 ± 0.6 among students. The instructors reported 8 ± 1.3 flight hours (hours/week), while the students reported 4 ± 0.7. The dominant upper limb of all participants was the right one.

**Figure 3:**
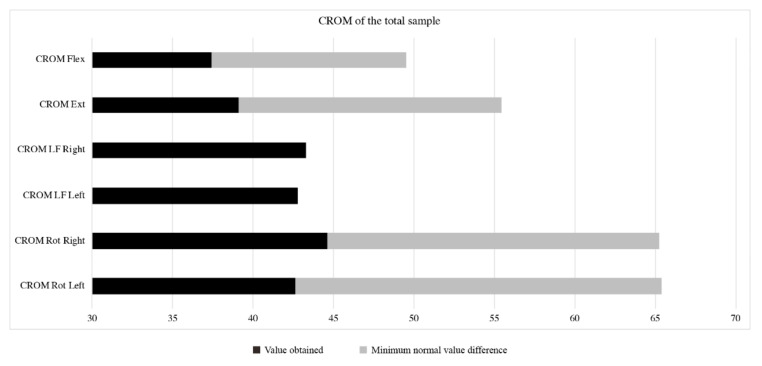
Cervical Range of Motion (CRoM) results.

The CRoM showed no statistically significant differences between the groups (*p* ≤ 0.05) (Table 1). However, limitations were observed in cervical flexion (37.4 ± 9.0), extension (39.1 ± 7.7), left rotation (42.6 ± 8.1) and right rotation (44.6 ± 6.6), with values lower than those reported as normalized values (maximal flexion: 49.52°; maximal extension: 55.46°; maximal rotation right: 65.26°; maximal rotation left: 65.38°) [27] (Figure 3). Specifically, six instructors and seven students scored below 43 degrees in the flexion parameter. Finally, the total sample had values less than 65° in the left and right rotations.

**Figure 4:**
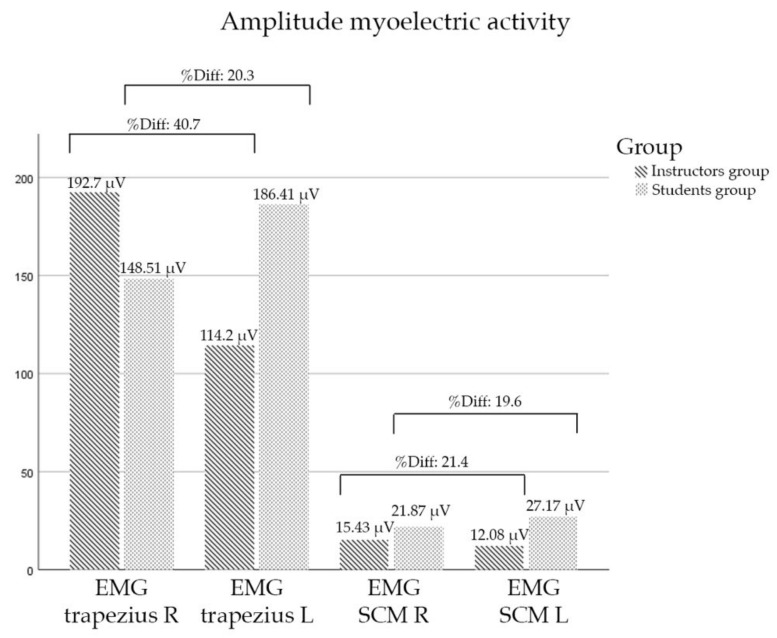
Amplitude of myoelectric activity and percentage difference for both groups. Abbreviations: EMG: electromyography; SCM: Sternocleidomastoid; R: Right; L: Left; µV: microvolts.

**Figure 5:**
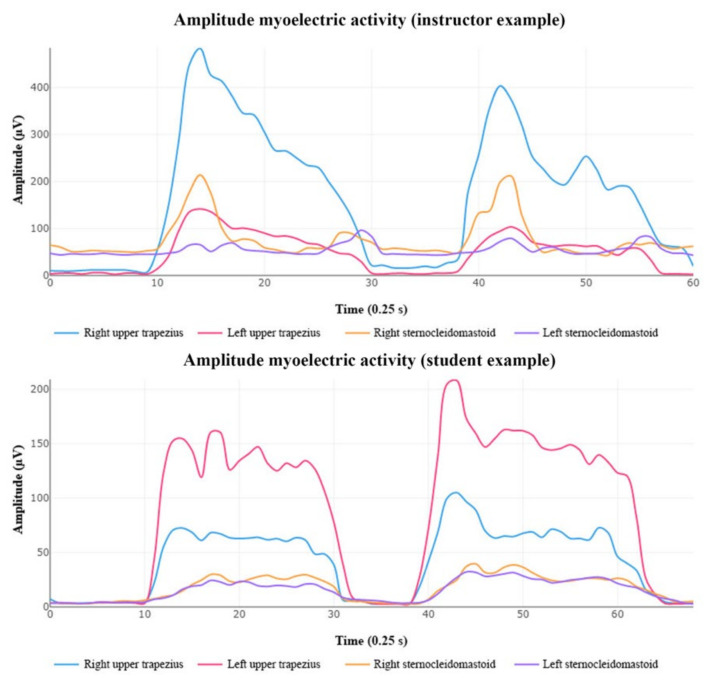
Example of amplitude differences of myoelectric activity detected between students (down) vs. instructors (up) represented by the right upper trapezius (blue line), left upper trapezius (red line), right sternocleidomastoid (yellow line) and left sternocleidomastoid (purple line) muscles.

In respect to myoelectric activity, no statistically significant differences were observed between the bilateral muscles or between the groups (Table 2). However, there were major changes in percentual differences of the upper trapezius muscles’ EMG amplitude in both groups. Upper trapezius of instructors: percentage difference of 40.7%; Upper trapezius of students: percentage difference of 20.3%; SCM of instructors: percentage difference of 21.4%; SCM of students: percentage difference of 19.6% (Figure 4 and Figure 5).

Table 3 shows a statistically significant correlation between the NDI and the age of pilots in both groups (β = 1.353, *p* < 0.01) (Table 3). Furthermore, the NDI showed a statistically significant correlation with the number of flight hours per week (β = 0.805, *p* = 0.003). In both cases, there was a positive relationship between the variables (age and number of flight hours increase with the perceived disability) (Table 3).

Table 4 shows a statistically significant correlation between the NDI and the CRoM left rotation (β = −0.880, *p* = 0.002) The relationship found that perceived disability was associated with a decrease in CRoM left rotation.

## 4. Discussion

The aim was to analyze biopsychosocial parameters of fighter pilots with flight-related neck pain. Our results are partially consistent with those from previous studies that have researched neck pain in military personnel from a biopsychosocial perspective [13,23,24,25,38]. This biopsychosocial approach could be reflected in the strong association found between the degree of cervical disability (psychological factor) and left cervical rotation (physical symptom). Previously, López-de-Uralde-Villanueva et al. [25] showed a strong association in infantry military personnel between fear of movement (kinesiophobia) and deficits in cervical flexion, lateral flexion, and rotations. These authors also observed strength deficits in cervical muscles as well as in cervical disability and in catastrophizing pain.

Perceived disability was higher in pilots who showed greater experience (number of flight hours) as well as in older ones. This parameter has been analyzed among military personnel [23,25]. Bahat et al. [23] showed mild pain and disability (VAS: 4.3 ± 2.3; NDI: 17.76 ± 9.6) in a sample size of 40 pilots with flight-related neck pain, and our results are similar to those reported in that study (VAS: 4.72 ± 1.84; NDI: 14 ± 4.41). This parameter showed a strong association with CRoM at the left rotation (Table 4). This fact could be explained by two reasons: (i) the full sample showed values lower than the cut-off points for the CRoM rotation in subjects with neck pain [27], which could have influenced the performance of the pilot’s cervical region, and (ii) according to Williams et al. [38], the range of movements obtained for flexion, lateroflexion and rotation are among the necessary ranges needed in order to execute flight tasks, which could minimize the degree of perceived disability, which was mild disability (28%) for the fighter pilots of this study.

Given this, we agree with Lange et al. [13] on the relevance of assessing the necks of fighter pilots to ensure optimal performance during technical operations in flights. The results could provide knowledge of the design of individualized training programs aimed at addressing the identified limitations.

### 4.1. Primary Outcomes

Although until now the adequate CRoM for flights has not been exactly defined, the CRoM for flight pilots has been mentioned [38]. The results showed limitations in inflexion, extension and rotation movements according to Kauther et al. [27]. Our results were consistent with previous studies that have found CRoM deficits among military personnel (e.g., air pilots) with neck pain [24,39]. Concretely, data reported during flights have shown that more time is being spent in mild flexion and twist-rotation postures [38]. Despite this, the CRoM is not the only parameter that determines the neck load and performance of flight pilots. Williams et al. [38] reported other factors such as time spent in mild and severe postures, cockpit position (pilot or co-pilot), as well as the number of flight hours and aircraft model.

In this sense, these authors showed that co-pilots spent more time than pilots in mild flexion, as well as in mild and severe twist-rotation postures, which was three times higher than for pilots. Regarding the limited CRoM parameters, our results differ from those shown by López-de-Uralde-Villanueva et al. [25] among military ground personnel, where restrictions in the CRoM were observed in the flexion and lateral-flexion parameters. These differences could be due to the characteristics of the military population that operates aircraft versus the military ground population. Recently, Williams et al. [38] indicated the existence of differences in posture depending on the posture in the cockpit. The fact that the instructors from our study were positioned in the back seat during the student’s instruction (fighter-attack Northrop F-5B) could be an influential factor in the loss of degree of movement.

### 4.2. Secondary Outcomes

PPT has been related to pain mechanisms in patients with neck pain. Among those, catastrophizing pain levels, kinesiophobia and cervical disability have been found. Walton et al. [40] obtained statistically significant associations between PPT and these variables. However, there is no clear evidence of its implication in fighter pilots. Concretely, Lange et al. [13] determined from fifty-five F-16 pilots that tenderness of either the right or the left levator scapulae and upper trapezius muscle were significantly indicative of self-reported neck pain within the last 3 months (*p* = 0.024 and *p* = 0.026, respectively). In our study, even though no statistically significant differences between groups nor association with other parameters was found despite obtaining values close to 3 Kg/cm2 (indicative of high sensitivity) [40] in both the levator scapulae and SCM muscles (Table 2). Future studies are needed to understand the impact of pain and discomfort in military pilots with flight-related neck pain.

Further studies are needed in order to consider PPT as an indirect indicator in the assessment of neck pain.

On the other hand, the myoelectric-activity assessment in fighter pilots has been studied in simulated flight conditions at different Gz forces [41]. However, there are no known studies to date that have specifically examined neck-muscle activity levels in relation to neck pain and injury thresholds in fighter pilots [3]. Previous studies showed significant changes between fighter and helicopter pilots with and without flight-related neck pain [31,42]. The technical differences between helicopters and fighter aircraft, as well as the movements and actions executed by the pilots (instructors vs. students) could again explain the differences in the demands on muscle groups (Figure 4 and Figure 5).

In this sense, the amplitude of the myoelectric activity in the right upper trapezius of instructors pilots (Figure 5) could be explained by: (i) the posture adopted by the right shoulder when controlling the thrust level in a fighter-attack Northrop F-5B and (ii) the muscle fatigue accumulated over years of flights by veteran pilots [31]. The authors agree with Farrel et al. [3] that the types of muscle contractions and myoelectric-activity responses are not indicative of an increased risk of injury in fighter pilots. These data could be interesting in aviation environments. Our results cannot be compared with those from other studies performed under the effect of G-forcer, nor with those in which the registry of data is made with an MVC higher than 50% [16]. Further studies are needed to analyze which MVC is better in order to assess flight-related neck pain, as well as environmental conditions (at rest or reproducing the cockpit’s conditions).

Regarding the catastrophizing pain, López-de-Uralde-Villanueva et al. [25] observed, among military ground personnel, higher values on the PCS scale in those participants who had higher levels of kinesiophobia. However, the values shown in both groups (with kinesiophobia, 10.77–6.82; without kinesiophobia, 5.4–5.29) were lower than those obtained in our study for both groups (group 1: 12.29 points; group 2: 16.91 points). Significant changes were achieved in these types of subjective measures after applying physical training programs and their combination with cognitive behavioral techniques, both in populations with NSMNP and in fighter pilots with neck and shoulder pain [13,43]. Further research would be promising due to the insufficient evidence among military personnel.

### 4.3. Practical Implications and Study Limitations

The obtained results are in line with other previous studies whose purpose was to analyze the health status of pilots in order to improve flight safety. According to the existing scientific literature, it seems necessary to obtain recommendations for optimizing the pilot’s performance from a biopsychosocial perspective. In this sense, Flynn et al. [44] recently reported the clinical benefits after physiotherapy among the military population with persistent pain. We believe that implementing a biopsychosocial-assessment approach such as the one proposed in this study could improve the impact of physiotherapy treatments in this population. Even though the obtained results provided relevant data, the reduced sample size and the unequal variances could possibly affect the statistical power and Type I error rates. Furthermore, all the participants were men. Future studies, with larger sample sizes, including pilots (males and females) from different air bases are needed to analyze other factors such as body-borne equipment, aircrew behaviors, or aircraft workspace.

## 5. Conclusions

The range of cervical movement (left rotation) has an influence on the degree of perceived disability in fighter pilots diagnosed with flight-related neck pain (instructors and students). This factor could be influenced by the number of flight hours and accumulated experience as an F-5 fighter pilot.

## Figures and Tables

**Table 1:** Baseline characteristics of sociodemographic characteristics and primary outcomes of participants (mean ± standard deviation and median ± quartiles).

	Total Sample (*n* = 18)	Instructor Group (*n* = 7)	Student Group (*n* = 11)	*p* Value *
Age (years)	26 ± 7.3	34 ± 6.1	21 ± 0.6	0.000 *
	22 ± 9	34 ± 7	22 ± 1	
Height (cm)	178 ± 7.1	177 ± 7.9	179 ± 6.9	0.93
	176.5 ± 11	176 ± 10	177 ± 12	
Weight (kg)	75.7 ± 9.7	78.4 ± 11.7	73.9 ± 8.3	0.57
	73.5 ± 14	82 ± 19	72 ± 6	
BMI (kg/m^2^)	23.7 ± 2.2	24.8 ± 2.5	23.0 ± 1.7	0.09
	23.38 ± 3.43	26.08 ± 4.11	23.12 ± 2.18	
Number of flight hours (hours/week)	5 ± 2.2	8 ± 1.3	4 ± 0.7	0.000 *
	4.5 ± 3.8	7.5 ± 2	4 ± 2	
Number of hours of physical activity (hours/week)	3 ± 1.9	4 ± 2.3	3 ± 1.3	0.13
	2.75 ± 2.5	3 ± 4	2 ± 1	
JPS (°)	5.7 ± 0.8	5.6 ± 0.8	5.7 ± 0.8	0.93
	5.9 ± 0.9	6 ± 1.02	5.83 ± 0.5	
VAS (cm)	4.72 ± 1.84	5.57 ± 1.99	4.18 ± 1.60	0.18
	4.5 ± 4	6.5 ± 5	5 ± 4	
NDI (0–50)	14 ± 4.4	16 ± 6.3	13 ± 2.0	0.15
	12 ± 4	15 ± 5	12 ± 2	
Cervical Range of Motion (CRoM)				
CRoM__Flex_ (°)	37.4 ± 9.0	34.9 ± 8.9	39.0 ± 9.1	0.42
	38 ± 14	34 ± 12	40 ± 18	
CRoM__Ext_ (°)	39.1 ± 7.7	36.3 ± 6.1	40.9 ± 8.3	0.25
	39 ± 15	35 ± 11	40 ± 19	
CRoM_Right_LF_ (°)	43.3 ± 6.2	42.3 ± 6.4	43.9 ± 6.4	0.72
	43.5 ± 8	43 ± 6	45 ± 12	
CRoM___Left_LF_ (°)	42.8 ± 7.0	41.0 ± 8.8	44.0 ± 5.7	0.66
	45 ± 9	45 ± 18	45 ± 8	
CRoM___Right _Rot._ (°)	44.6 ± 6.6	43.6 ± 5.8	42.3 ± 7.3	0.79
	42 ± 11	42 ± 6	42 ± 11	
CROM___Left_Rot_ (°)	42.6 ± 8.1	37.6 ± 7.1	45.9 ± 7.0	0.056
	44 ± 13	40 ± 11	47 ± 13	

Abbreviations: BMI: Body Mass Index; CRoM: Cervical Range of movement; JPS: Joint Position Sense; LF: Lateral Flexion; NDI: Neck Disability Index; VAS: Visual Analogue Scale.* Indicates between-groups statistical significance *p* ≤ 0.05.

**Table 2:** Baseline characteristics of secondary outcomes of participants (mean ± standard deviation and median ± quartiles).

	Total Sample (*n* = 18)	Instructor Group (*n* = 7)	Student Group (*n* = 11)	*p* Value *
Pressure Pain Threshold (PPT)				
PPT Trapezius Right (kg/cm^2^) (Active/Latent)	3.2 ± 0.7	3.2 ± 0.5	3.1 ± 0.8	0.60
	15.5 ± 1.2	3.5 ± 0.9	3 ± 1.5	
	(15/3)	(6/1)	(9/2)	
PPT Trapezius Left (kg/cm^2^) (Active/Latent)	2.8 ± 0.9	3.1 ± 0.8	2.7 ± 0.9	0.38
	2.6 ± 1.3	3.4 ± 1.4	2.5 ± 1.6	
	(17/1)	(7/0)	(10/1)	
PPT SCM Right (kg/cm^2^) (Active/Latent)	1.5 ± 0.6	1.7 ± 0.8	1.3 ± 0.3	0.18
	1.3 ± 0.4	1.4 ± 0.7	1.2 ± 0.3	
	(2/16)	(1/6)	(1/10)	
PPT SCM Left (kg/cm^2^) (Active/Latent)	1.5 ± 0.4	1.6 ± 0.5	1.4 ± 0.3	0.80
	1.3 ± 0.6	1.3 ± 0.8	1.3 ± 0.4	
	(3/15)	(1/6)	(2/9)	
PPT Levator scapulae Right (kg/cm^2^) (Active/Latent)	3.2 ± 0.9	3.0 ± 0.7	3.4 ± 1.0	0.86
	3 ± 1.2	3 ± 0	3 ± 1	
	(14/4)	(5/2)	(9/2)	
PPT Levator scapulae Left (kg/cm^2^) (Active/Latent)	3.2 ± 0.9	3.0 ± 0.5	3.3 ± 1.1	0.48
	3.1 ± 0.9	3 ± 0.8	3.1 ± 1.1	
	(15/3)	(7/0)	(8/3)	
Myoelectric Activity				
EMG Trapezius Right (µV)	165.7 ± 85.4	192.7 ± 84.1	148.5 ± 85.5	0.42
	178.5 ± 142.6	195.9 ± 116	161.6 ± 187.4	
EMG Trapezius Left (µV)	158.3 ± 112.8	114.2 ± 57.7	186.4 ± 131.9	0.37
	146.7 ± 160.8	143.3 ± 111.8	166.3 ± 213.2	
EMG SCM Right (µV)	19.4 ± 13.4	15.4 ± 8.7	21.8 ± 15.5	0.66
	13.4 ± 23.7	12.5 ± 12.5	14.3 ± 26.1	
EMG SCM Left (µV)	21.3 ± 17.3	12.1 ± 3.9	27.1 ± 20.1	0.06
	14.6 ± 13.4	11.2 ± 7.6	19.2 ± 36.6	
TAMPA (11–44)	30.7 ± 6.9	32 ± 6.9	30 ± 5.8	0.38
	31.5 ± 12	32 ± 13	29 ± 12	
PCS (0–52)	15 ± 8.8	12 ± 10.8	17 ± 7.3	0.21
	15.5 ± 13	14 ± 16	17 ± 8	

Abbreviations: EMG: electromyography; PCS: Pain Catastrophizing Scale; PPT: Pressure Pain Threshold; SCM: Sternocleidomastoid.* Indicates between-groups statistical significance *p* ≤ 0.05.

**Table 3:** Correlation level between NDI and anthropometric characteristics of the total sample.

	*P*	β	*p* Value
NDI	0.293	1.353	<0.01 *
Age (years)			
NDI	0.237	1.790	0.379
Height (cm)			
NDI	0.403	0.224	0.347
Weight (kg)			
NDI	0.332	0.227	0.203
BMI (kg/m^2^)			
NDI	0.702	0.805	0.003 *
Number of flight hours			
NDI	0.216	0.182	0.235
Number of hours of physical activity/week			

Abbreviations: NDI: Neck Disability Index, BMI: Body Mass Index, cm: centimeter, kg: kilograms, *P*: Spearman’s rank correlation coefficient, β: regression coefficient. * Indicates between-groups statistical significance *p* ≤ 0.05.

**Table 4:** Correlation level between NDI and CRoM of the total sample.

	*P*	β	*p* Value
NDI	–0.696	–0.167	0.374
CROM_Flex_ (°)			
NDI	–0.435	–0.077	0.749
CROM_Ext_ (°)			
NDI	–0.272	–0.477	0.196
CROM_LF_ Left (°)			
NDI	–0.206	–0.346	0.059
CROM_LF_ Right (°)			
NDI	–0.782	–0.880	0.002 *
CROM_Rot._Left (°)			
NDI	–0.348	–0.552	0.032 *
CROM_Rot._ Right (°)			

Abbreviations: NDI: Neck Disability Index, CRoM: Cervical Range of Motion, Flex: Flexion, Ext: Extension, LF: Lateroflexion; Rot: Rotation, *P*: Spearman’s rank correlation coefficient, β: Regression coefficient. * Indicates between-groups statistical significance *p* ≤ 0.05.

## Data Availability

We have not communicated any data and therefore exclude this statement.
